# Respect and political disagreement: Can intergroup respect reduce the biased evaluation of outgroup arguments?

**DOI:** 10.1371/journal.pone.0211556

**Published:** 2019-03-26

**Authors:** Silke Eschert, Bernd Simon

**Affiliations:** Institute of Psychology, Kiel University, Kiel, Germany; Rice University, UNITED STATES

## Abstract

Past research indicates that in political debates the same arguments are judged very differently depending on the perceiver’s own position on the issue, because positions on controversial issues are often tied to collective identities. In this article, we test the assumption that equality-based respect from an opposing opinion-based group can reduce such biases. Results confirmed that identification as an opponent or proponent of a contested issue was negatively related to evaluations of outgroup arguments (Study 1) and that this negative link was no longer significant when intergroup respect was experimentally induced (Study 2). Results support the notion that disagreements over political issues are intergroup conflicts, in which different socio-political groups struggle for recognition, and that approaches that protect collective identities and improve intergroup relations should be employed to de-escalate them.

## Introduction

In debates over political disagreements, one central problem is the biased processing of political arguments or other issue-related information. Social psychologists have long observed that people tend to seek out information supporting their political opinions and tend to avoid disconfirming information [1,2]. Once people are exposed to disconfirming information, however, it is subjected to critical scrutiny and very likely judged as unconvincing, whereas confirming information is accepted at face value [2–4]. Consequently, ambiguous or mixed information tends to be interpreted in a way that confirms pre-existing opinions and an exchange of arguments often does not serve to reduce conflict but instead leads to greater polarization [2,4,5]. Finding ways to reduce, or even remove, these biased evaluations may, therefore, be crucial for constructive political discourse.

While these phenomena are most often researched on the level of individual opinions, it is important to note that public disagreements over political issues are often best understood as conflicts between different societal, ideological or opinion-based groups. A contested issue like kosher butchering, for example, is typically not debated between individuals, but most likely between members of certain religious groups on one side and proponents of animal rights (i.e., an opinion-based group) on the other side.

### Political disagreements as intergroup conflicts

In fact, a wealth of research shows that dynamics of intergroup conflict are present in political disagreements. Especially in the US, where political polarization appears to be intense, members of different political groups (i.e., Republicans and Democrats) display open animus and discriminate against opposing partisans, to a degree that exceeds discrimination based on ethnicity [6,7]. Bliuc and her colleagues [8] could also show that both believers and sceptics of climate change in the US identify and act as members of distinct groups. In this study, collective action intentions (e.g., intentions to donate money or to attend political rallies) were predicted by ingroup identification, anger at the opposing group, and collective efficacy; that is, collective action in this political context was motivated by the same psychological variables as in other intergroup contexts [9].

Further, the biased evaluation of opposing political arguments can also be explained by intergroup processes. Identified group members are motivated to evaluate information negatively that threatens their collective identities; even more so when they are strongly identified group-members [10]. Outgroup arguments may be threatening when they imply that the ingroup’s convictions are false, inferior, or even morally objectionable (creating group esteem threat [11]) or when they call the ingroup’s identity-defining opinions or values into question (creating symbolic threat [12,13]).

This was also shown by Nauroth and his colleagues [14,15]. In these studies, gamers in Germany were confronted with scientific reports on the negative consequences of violent video games. Nauroth and colleagues [14] found that identification with the group of gamers predicted fundamentally negative evaluations of scientific reports demonstrating detrimental effects of playing violent video games. Identification also predicted these biased evaluations over and above individual-level predictors, such as attitude inconsistency or gaming habits. Nauroth and colleagues [15] could further show that identification with the group of gamers was associated with posting discrediting online comments on such scientific reports. Moreover, the authors could show that these reactions could be alleviated when participants’ collective identity was buffered against threat. In this study [15], participants completed an anagram solving task. Afterwards, in the collective affirmation condition, they received positive group-based feedback (i.e., feedback saying that gamers usually score high on these tests). For collectively affirmed participants, stronger identification did no longer predict more discrediting commenting.

This research demonstrates that in order to build constructive political discourse exchanging arguments will not suffice, but researchers and practitioners will instead need to focus on the dynamics of intergroup conflict and on strategies known to reduce such conflicts [8]. In the following, we will argue that explicitly treating political opponents with respect is such a strategy. Intergroup respect has the potential to reduce intergroup bias and to improve intergroup relations and, thus, also to facilitate a more constructive political debate.

### Respect in intergroup contexts

To clarify the concept of respect, we draw on Honneth’s influential recognition theory [16]. Honneth theorized respect to be based primarily on equality recognition and recent empirical findings support this claim [17,18]. Simon and Grabow [17] focused on the gay and lesbian community in Germany and found that the experience of being respected in society primarily reflected perceived recognition of gays and lesbians as equal members of society. In their study, perceived respect from the Muslim community was also negatively related to anti-Muslim attitudes among gay and lesbian participants and, thus, to improved intergroup relations. This is consistent with research conducted by Huo and Molina [19] who found a negative relationship between perceived societal respect and ingroup favoritism among different ethnic groups in the US.

Huo and Molina [19] argued that respect can reduce ingroup favoritism and improve intergroup relations because members of respected groups have less psychological need to show ingroup favoritism as a means to defend their collective identity. The authors explain this based on work on intragroup respect which suggests that respectful treatment communicates the individual’s value to the group [20]. In an intergroup context, respectful treatment should communicate the ingroup’s value to another group or to society at large and, hence, affirm the ingroup’s standing and render ingroup favoritism unnecessary. In line with this, Simon and Schaefer [21,22] demonstrated that disapproved outgroups can be tolerated when they are respected and when others feel respected by them.

However, the difference between equality-based respect and group affirmation (i.e., positive achievement-based feedback [15]) or status affirmation [23] should be clarified. While other studies have affirmed high achievement or high status (i.e., higher than for others), we treat respect as the recognition of equal status. This is an important theoretical distinction and it has practical implications, too. While recognizing the high or higher status of an opposing group in a political debate might be difficult, recognizing equality should be comparably less costly. When, for example, established democratic political parties are faced with populist or radical right-wing parties, recognizing their achievements or their high status might be a lot more costly (or even painful) than recognizing their democratic right to present their arguments, to be heard out and to be taken seriously just like any other political party.

In summary, when public disagreements over political issues are understood as intergroup conflicts, in which different socio-political groups struggle for recognition, it is reasonable to assume that respectful treatment coming from an opposing group should serve to reduce (or even remove) the biased evaluation of the opposing groups’ arguments. Therefore, intergroup respect could lay the foundation for a more constructive political discourse in the face of disagreement.

## The present research

To create a credible intergroup context we needed a contested issue, for which there would be substantial numbers of both proponents and opponents in our student samples. A pretest was conducted to find a suitable political issue. A correlational study (Study 1) was then conducted to investigate if argument evaluation depended on participants’ position on the issue and the strength of their identification as either proponent or opponent. Based on previous research [10,14,15], a negative link between ingroup identification and the evaluation of outgroup arguments was expected (Hypothesis 1). Study 2 was designed to test the effect of experimentally induced intergroup respect or disrespect on the evaluation of outgroup arguments. In accordance to the findings of Nauroth and colleagues [15], we assumed that the negative link between ingroup identification and the evaluation of outgroup arguments should be reduced in the respectful condition (Hypothesis 2).

The Ethics Commission of the Medical Faculty of the Christian-Albrechts-Universität zu Kiel determined the study to be exempt from the requirement for ethical review. However, all studies were conducted in full accordance with the declaration of Helsinki, and with the ethical guidelines of the German Association of Psychologists (DGPs) and the American Psychological Association (APA). This includes–among others–obtaining informed consent, the right to withdraw at any time, and data protection. Therefore, before starting the study, detailed information regarding ethical guidelines were provided. Participants were informed that they could easily withdraw from the study at any time by closing the internet browser (Pretest, Study 1) or by leaving their cubicle (Study 2), that all their data would be analyzed anonymously and that collected e-mail addresses would be saved in a separate file. Contact details of the first author were provided in all questionnaires. Signed consent forms were collected in our laboratory study (Study 2); in our online studies (Pretest, Study 1), participants ticked a box to indicate that they understood the instructions and consented to participate in the study. No deception was involved in the pretest or Study 1. In Study 2, participants were fully debriefed about the experimental manipulation, in order to give them the chance to withdraw their consent on that basis, which no one did.

## Pretest

A pretest (*N* = 92) was conducted to find a political issue that provoked sufficient disagreement in student samples and for which importance and identification would vary among participants. In a short questionnaire participants were asked to indicate their position on ten different political issues that we assumed were relevant to students and also debated among students. Participants were also asked to indicate how important their own position on the issue was to them personally. The surveillance of social networks by security agencies was identified as a suitable contested issue. Out of 92 participants 39 indicated that they were proponents, 29 indicated that they were opponents and 11 were undecided (13 participants failed to complete the items on this issue). Further, suitable variance was found for ratings of personal importance (*M* = 4.77, *SD* = 1.39; rated on a 7-point Likert scale).

## Study 1

In Study 1, both proponents and opponents of the surveillance of social networks were presented with pro and con arguments concerning this issue. Thus, the aim of Study 1 was to test the assumption that individuals with different positions on the issue would evaluate the same arguments differently and that these different evaluations also depended on the strength of ingroup identification. A negative link between ingroup identification and the evaluation of outgroup arguments was expected (Hypothesis 1).

### Method

#### Participants

One hundred and three undergraduate students from Kiel University (64 women, 38 men, 1 person who did not indicate their sex; *M*_age_ = 22.9 years, SD = 4.6 years, range 18–45 years) participated in the study. The students were enrolled in various programs; students of psychology were not invited to participate. Concerning their position on the issue, 65 participants stated that they were proponents and 38 participants stated that they were opponents of surveillance. The study was conducted online; participants were contacted through social network groups of their university. All participants could take part in a raffle for book vouchers worth 25 Euro (approx. 27.7 USD).

#### Procedure and measures

Participants first read about the contested issue at hand. The question read as follows: “Should national security agencies have access to messages from social networks?” Participants then stated their position on the issue and their degree of identification as either opponent or proponent. Subsequently, all participants rated the strength of 18 pro and 18 con arguments. To avoid order effects, half of all proponents and half of all opponents received pro arguments first, whereas the respective other half received con arguments first.

Participants stated their position on the issue on a 10-point scale ranging from -5 *strongly against* to +5 *strongly in favor* of security agencies having access to these messages. Ingroup identification was assessed with three items adapted from the scale by Leach and colleagues [24]. The items read as follows: (1) “I see myself as a proponent / opponent”, (2) “I feel a bond with others, who also support / oppose surveillance” and (3) “Being a proponent / opponent is an important part of how I see myself”. Items were answered on a 7-point Likert scale ranging from 1 *do not agree at all* to 7 *fully agree*. The scale was reliable with Cronbach’s α = .70 for proponents and Cronbach’s α = .80 for opponents. Mean identification scores were close to the scale midpoint for both proponents (*M* = 3.85, *SD* = 1.06) and opponents (*M* = 3.95, *SD* = 1.58). While a similar number of men proposed / opposed surveillance (21 proponents, 17 opponents), more women proposed it (44 proponents, 20 opponents), although this difference did not reach significance, *χ*²(1) = 1.88, *p* = .171. However, sex was correlated with argument ratings, *r* = .29 (*p* = .003), with women rating arguments more favorably (*M* = 4.53, *SD* = 0.61) than men (*M* = 4.14, *SD* = 0.41; *t*(98,60) = -3.89, *p* = .001, *d* = 0.76). Therefore, sex was controlled in subsequent analyses.

Pro and con arguments were about 20 to 30 words long. A sample con argument read as follows: “Governments have no control over their intelligence services. Therefore, there will always be misuse of data and a lack of transparency.” A sample pro argument was: “Privacy is important but the war on terror and criminal investigations are more important.” Argument strength was rated on a 7-point Likert scale ranging from 1 *weak argument* to 7 *strong argument*. Argument ratings were reliable with Cronbach’s α = .90 for pro arguments and Cronbach’s α = .88 for con arguments (for descriptive statistics see also Table A, in S1 Appendix).

### Results

A mixed-model ANOVA was conducted using SPSS linear mixed-models. Argument position (pro vs. con) was included as a within-subjects-factor and participant position (proponent vs. opponent) as a between-subjects factor. Ingroup identification was centered and used as a continuous moderator.

**Fig 1:**
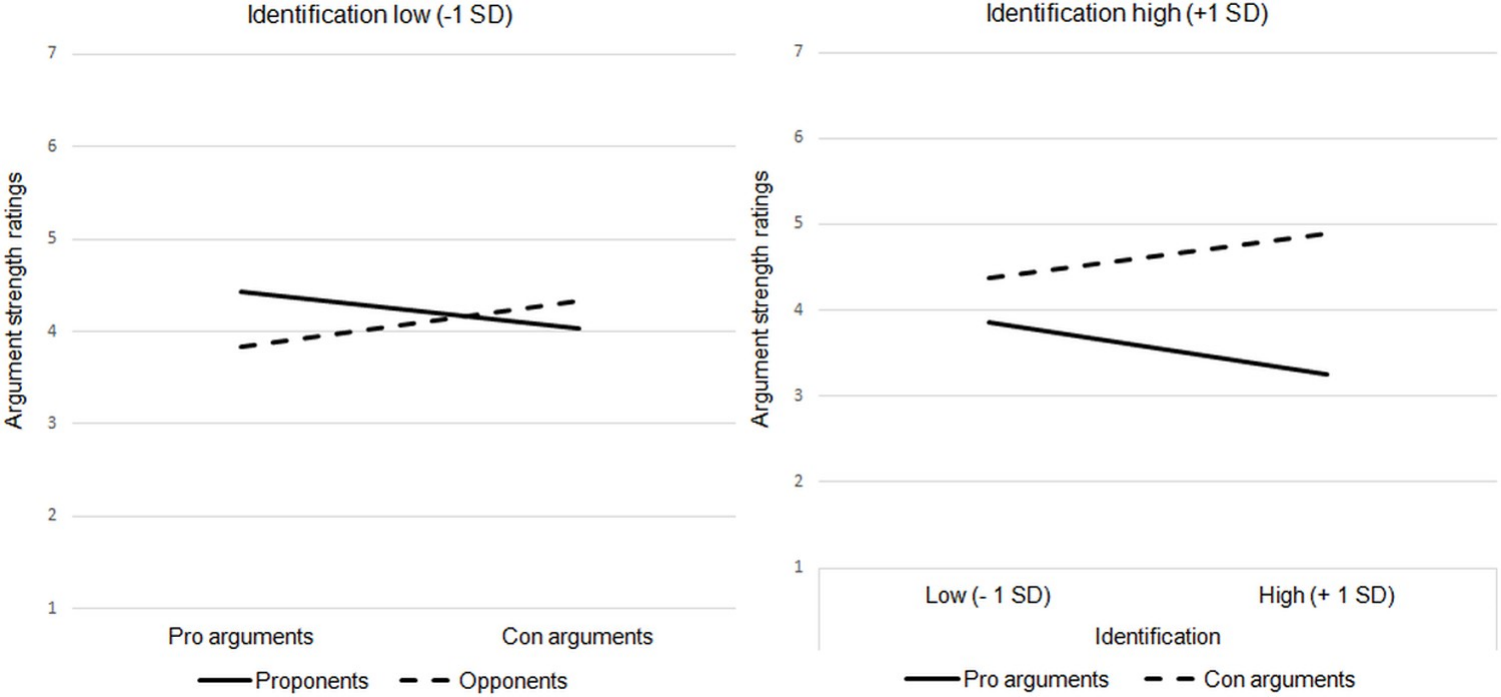
Perceived argument strength as a function of participant position, argument position and ingroup identification (Study 1).

No main effect of argument position could be observed, *F*(1,99) = 0.08, *p* = .784, *ηp²* < .001, that is, pro arguments were not generally perceived to be stronger than con arguments or vice versa. There were no main effects for participant position (*F*(1,97.03) = 2.45, *p* = .121, *ηp²* = .02) or ingroup identification (*F*(1,97) = 0.35, *p* = .852, *ηp²* = .003), either. More important, a significant argument position by participant position interaction emerged, *F*(1,99) = 68.33, *p* < .001, *ηp²* = .41, indicating that for proponents pro arguments (*M* = 4.81, *SE* = 0.21) were stronger than con arguments (*M* = 3.66, *SE* = 0.22; *p* < .001), whereas for opponents pro arguments were weaker (*M* = 3.52, *SE* = 0.22) than con arguments (*M* = 4.60, *SD* = 0.23; *p* < .001). However, this two-way interaction was qualified by a significant argument position by participant position by ingroup identification interaction, *F*(1,99) = 25.58, *p* < .001, *ηp²* = .21. No other interaction was significant (all *p*s > .46; see also Table B in S1 Appendix). To decompose this three-way interaction, we ran our mixed-model ANOVA again for high and for low ingroup identification. To do this, ingroup identification was re-centered at one standard deviation above and below the mean (see also Table C and Table D in S1 Appendix). The analysis revealed that the argument position by participant position interaction was significant for lower (*F*(1,99) = 5.67, *p* = .019, *ηp²* = .05) and for higher ingroup identification (*F*(1,99) = 89.76, *p* < .001, *ηp²* = .47). However, the effect was clearly more pronounced for opponents and proponents with stronger ingroup identification. Weakly identified opponents evaluated con arguments only marginally more favorable (*M* = 4.34, *SE* = .27) than pro arguments (*M* = 3.83, *SE* = .26; *p* = .074) and for weakly identified proponents there was no observable difference in their evaluation of pro and con arguments (*M* = 4.43, *SE* = .24 versus *M* = 4.04, *SE* = .25; *p* = .124; see Fig 1, left panel). For highly identified proponents, on the other hand, pro arguments (*M* = 5.19, *SE* = .25) were quite clearly stronger than con arguments (*M* = 3.29, *SE* = .26; *p* < .001) and for highly identified opponents con arguments were quite clearly stronger than pro arguments (*M* = 4.86, *SE* = .24 versus *M* = 3.22, *SE* = .23; *p* < .001; see Fig 1, right panel). A stronger ingroup identification was also correlated with more negative strength ratings for outgroup arguments (*r* = -.33, *p* = .007 for proponents / con arguments and *r* = -.39, *p* = .017 for opponents / pro arguments) and with more positive strength ratings for ingroup arguments (*r* = .39, *p* = .001 for proponents / pro arguments and *r* = .33, *p* = .042 for opponents / con arguments).

### Discussion

Study 1 demonstrated that argument strength of pro and con arguments was not perceived as different per se but that evaluation depended on participants’ own position on the issue and the strength of their identification with the ingroup. The greatest differences in the evaluation of pro and con arguments was observed for strongly identified group members. Consistent with previous literature, there was a negative bias against outgroup arguments (confirming Hypothesis 1). In Study 2, we focused on these negative evaluations. Indicating a possible limitation, several participants commented on the ambiguity in the question about security agencies using “messages” from social networks. They stated that they would not be opposed to security agencies using public messages as long as private messages were not accessed. This might also explain the surprisingly large number of proponents in Study 1. To address this, the contested issue was clarified in Study 2.

## Study 2

In Study 2, outgroup (dis)respect was experimentally induced. As expected, in Study 1 we found a negative link between ingroup identification and the perceived strength of the respective outgroup’s arguments. In Study 2, we expected that this negative bias should be weakened or even removed when respect was received from the outgroup (Hypothesis 2).

### Method

#### Participants and design

One hundred and forty-six undergraduate students from Kiel University participated in the study. Nine participants stated that they were not active in any social network. These participants were excluded from further analyses. The final sample thus consisted of 137 students (68 women, 68 men, 1 person who did not indicate their sex; *M*_age_ = 22.8 years, *SD* = 2.8 years, range 19–32 years). Out of these 137 participants, 56 self-identified as proponents and 81 as opponents of the surveillance of social networks. Both opponents and proponents were randomly assigned to a high respect or low respect experimental condition. Ingroup identification was measured and used as a continuous moderator.

#### Procedure

The alleged purpose of the study was to collect and discuss different arguments concerning a controversial political issue. For each experimental session, up to eight students were invited to the laboratory, where they were seated at individual computer terminals in separate cubicles. Informed consent was obtained from all participants. As in Study 1, participants first read about the contested issue (i.e., surveillance of social networks). To resolve the ambiguity noted above, for Study 2 the contested issue was rephrased as follows: “Should national security agencies have access to all messages from social networks (public and private messages)?”. Participants indicated their position on the issue on the same scale that was used in Study 1 and were then asked to write down arguments in support of their position. This was done to increase the credibility of the cover story that there would be a discussion later. Next, ingroup identification was measured, also using the same items as in Study 1 (Cronbach’s α = .74 for opponents and α = .66 for proponents). Mean identification scores were again close to the scale midpoint for both groups. However, opponents reported a higher degree of identification (*M* = 4.28, *SD* = 1.14) than proponents (*M* = 3.81, *SD* = 1.08) in Study 2, *t*(135) = 2.40, *p* = .018, *d* = .42. In Study 2, a similar number of women proposed / opposed surveillance (30 proponents, 38 opponents), whereas more men opposed it (26 proponents, 42 opponents), although, again, the difference was not significant, *χ*²(1) = 0.49, *p* = .486. However, sex was again correlated with the dependent variable, *r* = .34 (*p* < .001). As in Study 1, women rated arguments more favorably (*M* = 4.22, *SD* = 0.88) than men (*M* = 3.58, *SD* = 0.89; *t*(134) = -4.24, *p* < .001, *d* = .73). Therefore, sex was again controlled in subsequent analyses.

Afterwards, participants read the respect manipulation, completed our manipulation checks and rated the strength of only twelve outgroup arguments. Argument ratings were reliable with Cronbach’s α = .73 for pro arguments and Cronbach’s α = .82 for con arguments. All arguments were taken from the pool of arguments from Study 1. However, the number of arguments had to be reduced to avoid a lengthy questionnaire. Finally, participants were fully debriefed and received 5 € (approx. 5.6 USD) for their participation.

To manipulate equality-based respect, participants were informed that, while they had been working on another task, the members of the participant’s outgroup had allegedly indicated their (dis)agreement with two statements concerning the intended treatment of the participant’s ingroup. These two statements read as follows: “the opinion of [ingroup] shall be discussed equally in the upcoming group discussion” and “the [ingroup] and their opinion shall be taken seriously”. In the high respect condition, the pooled answers of the outgroup members indicated agreement with these two statements, while in the low respect condition pooled answers indicated disagreement with both statements. Similar manipulations were already used effectively in past research [25].

As manipulation check, perceived equality recognition was measured both with regard to the individual participant (“I feel treated equally”, “I feel treated like a person of equal worth”) and for the entire ingroup (“my group is taken seriously”, “my group is treated equally”). This was done for exploratory reasons. In past research, participants usually received individual respect, not group-based respect. All items were answered on 7-point Likert scales ranging from 1 *do not agree at all* to 7 *fully agree*. Both measures were reliable with Cronbach’s α = .96 for individual treatment and with Cronbach’s α = .95 for treatment of the group (for descriptive statistics see also Table E, in S2 Appendix).

### Results

#### Manipulation checks

A two-way ANOVA was conducted with participant position (proponent vs. opponent) and respect (low vs. high) as between-subjects factors. For individual equality recognition a significant main effect emerged for the respect manipulation, *F*(1,133) = 235.25, *p* < .001, *η*_p_² = .64, indicating that perceived equality recognition was stronger in the high respect condition (*M* = 5.92, *SE* = .13) than in the low respect condition (*M* = 3.07, *SE* = .13). There was no effect of participant position, *F*(1,133) = 1.90, *p* = .171, *η*_p_² = .01, and no significant respect x participant position interaction, *F*(1,133) = .003, *p* = .955, *η*_p_² < .001. Results were almost the same for perceived group equality recognition. Participants in the high respect condition felt that their group was recognized as equal (*M* = 5.90, *SE* = .13), more so than participants in the low respect condition (*M* = 2.87, *SE* = .13), *F*(1,133) = 280.90, *p* < .001, *η*_p_² = .68. Again, there was no significant effect of participant position, *F*(1,133) = 3.42, *p* = .067, *η*_p_² = .03 and no significant interaction, *F*(1,133) < .001, *p* = .988, *η*_p_² < .001 (see also Table F, in S2 Appendix).

#### Argument ratings

**Fig 2:**
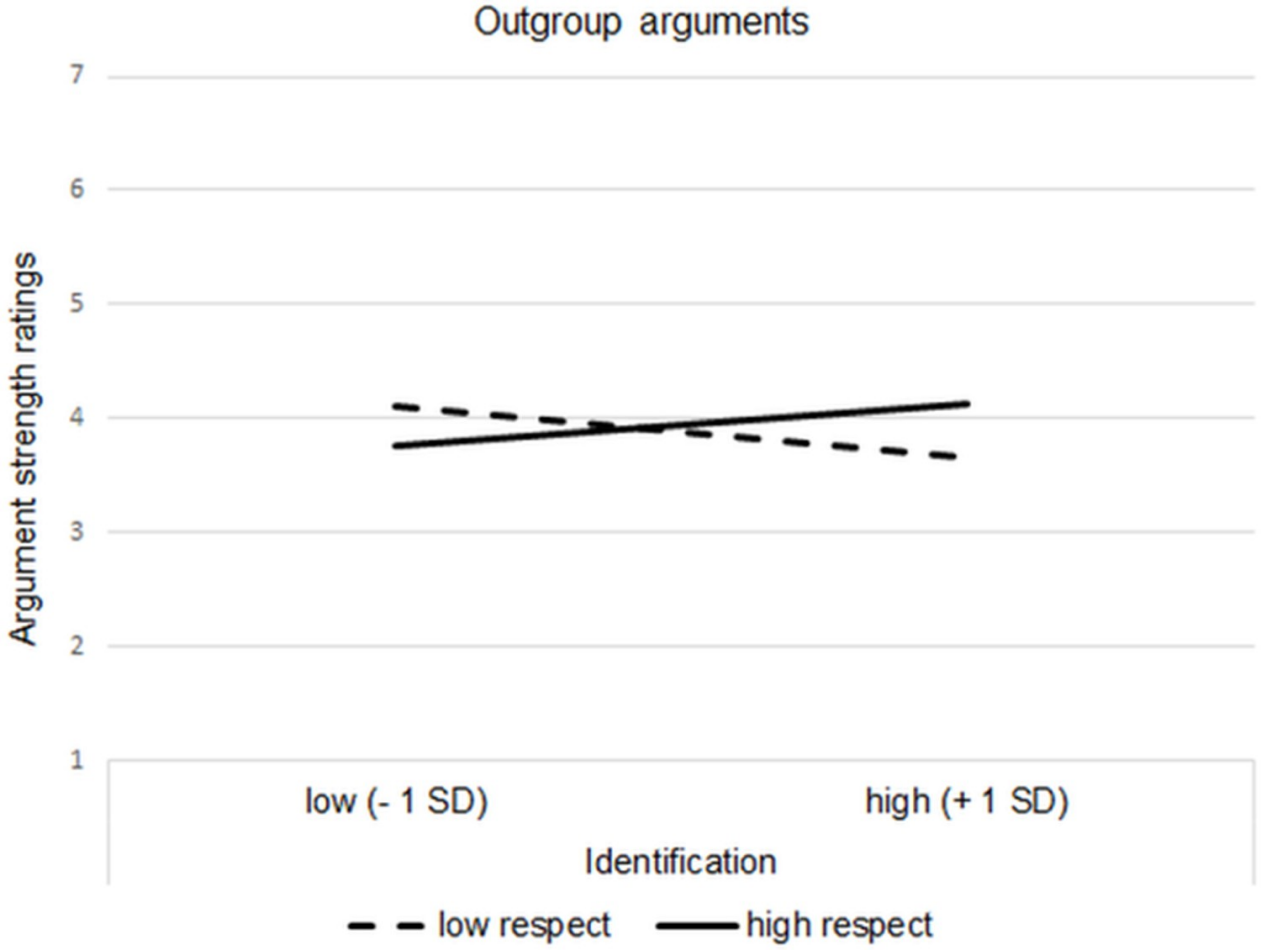
Perceived strength of outgroup arguments as a function of the respect manipulation and participants’ ingroup identification. **(Study 2)**.

Multiple linear regression was calculated to predict argument ratings based on participant position, induced respect and ingroup identification. Ingroup identification was centered and entered into the analysis as a continuous moderator as recommended by Aiken and West [26]. Tests for multicollinearity indicated that a very low level of multicollinearity was present (all *VIF*s < 1.22). None of the main effects were significant–neither for participant position, *B* = .08, *SE* = .08, *p* = .289, nor for the respect manipulation, *B* = .03, *SE* = .08, *p* = .707, nor for ingroup identification, *B* = -.02, *SE* = .07, *p* = .832. More important, as hypothesized, a significant respect x ingroup identification interaction emerged, *B* = .18, *SE* = .07, *p* = .016 (see Fig 2). No other interaction reached significance (all *p*s < .210; see also Table G, in S2 Appendix). Simple slope analysis of the respect x ingroup identification interaction revealed that, as hypothesized, ingroup identification was negatively related to the perceived strength of outgroup arguments in the low respect condition, *B* = -.19, *SE* = .10, *p* = .044. In the high respect condition, there was no significant relationship between ingroup identification and perceived strength of outgroup arguments anymore; results actually hinted at a trend in the opposite direction, *B* = .16, *SE* = .11, *p* = .144.

### Discussion

Study 2 demonstrated that the usually observed negative bias against outgroup arguments disappeared when intergroup respect was experimentally induced. While there was a negative link between ingroup identification and the evaluation of outgroup arguments when participants had received disrespectful treatment by the outgroup, this link was not significant anymore when participants had received respectful treatment by the outgroup (supporting Hypthesis 2). Notably, this effect was not qualified by participant position. No main effect of the respect manipulation was observed, either. However, we did not hypothesize that arguments would generally receive more positive ratings under high respect. We hypothesized that the negative bias should be reduced or even removed under high respect.

## General discussion

We started this article by pointing out the problematic role that the biased evaluation of outgroup arguments may play in public debates on political disagreements. While deliberation research (as conducted in the political sciences [27] would propose that an exchange of arguments should lead to depolarization, social psychological research has repeatedly shown that arguments from an outgroup source may be perceived as threatening if they contain negative information about the ingroup (e.g., that the ingroup is wrong or inferior [10,11]). To protect a threatened collective identity, those outgroup arguments are often avoided or judged as unconvincing [2–4]. As a result, an exchange of arguments may lead to greater polarization [2,4,5].

Prior research has already shown that higher identification with a group predicts more negative evaluations of information that may threaten the ingroup [10,14,15]. The present research replicates this finding in the context of political arguments. Moreover, the present research demonstrates that a concept that was imported from intergroup research, namely equality-based intergroup respect, may reduce or even remove this bias. Therefore, the present research has practical implications for de-escalating political debates and there are important theoretical implications as well.

Recent research has called for a change of perspective regarding public disagreements over issues like climate change [8] or media violence [14,15]. Disagreements like this are typically best explained as intergroup conflicts, in which collective identities may be threatened and in which different groups fight for respected collective identities; that is, as politicized struggles for recognition. Presenting scientific facts or exchanging arguments will not suffice to reach compromise between highly identified partisans from opposing parties. Instead, to facilitate a less defensive debate, procedures need to be employed that protect collective identities and improve intergroup relations [8, 28]. In this paper, we argued that intergroup respect should be a very useful concept for these purposes. And we would also like to highlight that respect and self-affirmation or collective affirmation are really not the same. While Nauroth and colleagues [15] and Bendersky [23] have demonstrated comparable effects using achievement-based group affirmation and group status affirmation, respectively, respect highlights equal treatment and equal status not high or higher status; nor does it require liking or agreement with any position of the other party, either [21, 22]. Equality-based respect is unlikely to enhance group esteem and more likely to serve a protective function. This is an important theoretical distinction. Further, the present research supports the notion that recognizing equality is sufficient to pacify conflict and that enhancement or recognizing superiority (e.g., better achievements than others) is unnecessary. In this sense, we can also theorize that public disagreements are struggles for equality, not superiority. And there are important practical implications as well. As Bendersky [23] already noted classic self-affirmation (or achievement feedback) manipulations are not actually feasible ways to de-escalate conflicts. In a real-world argument telling one conflict party to “go affirm yourself” (p. 164) is unrealistic and as we have noted above communicating high status might be costly or even painful. Instead, it should be easier to communicate that the opposing party is considered an equal conversation partner and that their arguments will be taken seriously. .

Despite the theoretical and practical relevance of the present research, some limitations to the present studies need to be addressed as well. While the surveillance of social networks is a controversial issue and motivated devaluation of outgroup arguments was observed, it is not as much a political hot topic as, for example, the refugee crisis in Europe or reproductive rights in the US. Also, surveillance may be less likely to be perceived as a moral issue. This might be relevant because research on moral conviction has shown that aspects of procedural justice seem to be less effective when attitudes are tied to moral convictions [29,30]. If intergroup respect is effective also in conflicts that more directly involve moral conviction, is an interesting question for future research.

Another topic of future research concerns the effect of intergroup respect on outgroup attitudes in the context of social and political disagreement. Past research has also shown that it is not only outgroup arguments that may be devaluated in the face of threatened collective identities but that this may affect attitudes towards members of the outgroup as well. Attacks to a group’s esteem may evoke greater perceived ingroup homogeneity, greater ingroup favoritism and also greater ingroup-outgroup differentiation and outgroup derogation [31–33], leading to deteriorating intergroup relations [34]. Since respect is related to positive intergroup relations [17] and respect was found to facilitate the identification with a common ingroup identity [25], respect could have a positive effect on outgroup attitudes in socio-political debates. This is especially relevant because research in the apparently very polarized US context has observed an increase in both the number of highly identified partisans and the social distance between these opposing partisans through recent decades [35, 36].

In summary, viewing debates on political disagreements from an intergroup perspective greatly contributes to our understanding of the possible escalating or de-escalating processes present in these debates [37]. This is not to say that in public disagreements people do not also fight for their convictions or that conflict would disappear as soon as the groups involved communicate respect for each other, but de-escalating conflict and reducing intergroup bias should remove some barriers to constructive political discourse. The present research supports the notion that removing such barriers should lead to less devaluation of what the opposing group has to say and makes compromise more attainable and that, thus, equality-based respect should be considered as a relevant construct in the literature on ideological or intergroup conflict. We certainly hope that the present research will stimulate further research in this area.

## Supporting information

S1 Appendix Additional tables for results section of Study 1. In this pdf document results for Study 1 are presented in tables.
(PDF)

S2 Appendix Additional tables for results section of Study 2. In this pdf document results for Study 2 are presented in tables.
(PDF)

## References

[1] Jonas E, Graupmann V, Fischer P, Greitemeyer T, Frey D. Schwarze Kassen, weiße Westen? Konfirmatorische Informationssuche und -bewertung im Kontext der Parteispendenaffäre der CDU. Zeitschrift für Sozialpsychologie. 2003;34(1):47–61.

[2] Taber CS, Lodge M. Motivated skepticism in the evaluation of political beliefs. Am J Pol Sci. 2006;50(3):755–69.

[3] Liberman A, Chaiken S. Defensive processing of personally relevant health messages. Personal Soc Psychol Bull. 1992;18(6):669–79.

[4] Lord CG, Ross L, Lepper MR. Biased assimilation and attitude polarization: The effects of prior theories on subsequently considered evidence. J Pers Soc Psychol. 1979;37(11):2098–109.

[5] Wojcieszak M, Price V. Bridging the divide or intensifying the conflict? How disagreement affects strong predilections about sexual minorities. Polit Psychol. 2010;31(3):315–39.

[6] Brandt MJ, Reyna C, Chambers JR, Crawford J, Wetherell G. The ideological-conflict hypothesis: Intolerance among both liberals and conservatives. Curr Dir Psychol Sci [Internet]. 2014;23(1):27–34. Available from: http://www.ssrn.com/abstract=2225989

[7] Iyengar S, Westwood SJ. Fear and Loathing across Party Lines: New Evidence on Group Polarization. Am J Pol Sci. 2015;59(3):690–707.

[8] Bliuc A-M, McGarty C, Thomas EF, Lala G, Berndsen M, Misajon R. Public division about climate change rooted in conflicting socio-political identities. Nat Clim Chang [Internet]. 2015;5:226–9. Available from: http://www.nature.com/doifinder/10.1038/nclimate2507

[9] van Zomeren M, Postmes T, Spears R. Toward an Integrative Social Identity Model of Collective Action: A Quantitative Research Synthesis of Three Socio-Psychological Perspectives. Psychol Bull. 2008;134(4):504–35. 10.1037/0033-2909.134.4.504 18605818

[10] De Hoog N. Processing of social identity threats: A defense motivation perspective. Soc Psychol (Gott). 2013;44(6):361–72.

[11] Branscombe NR, Ellemers N, Spears R, Doosje B. The context and content of social identity threat In: Ellemers N, Spears R, Doosje B, editors. Social identity: Context, commitment, content. Oxford, UK: Blackwell; 1999 p. 35–58.

[12] Stephan WG, Renfro CL. The role of threat in intergroup relations In: Mackie DM, Smith ER, editors. From Prejudice to Intergroup Emotions: Differentiated Reactions to Social Groups. New York: Psychology Press; 2002 p. 191–207.

[13] Stephan WG, Stephan CW. An integrated threat theory of prejudice In: Oskamp S, editor. Reducing prejudice and discrimination. Mahwah, N.J.: Psychology Press; 2000 p. 23–45.

[14] Nauroth P, Gollwitzer M, Bender J, Rothmund T. Gamers Against Science: The Case of the Violent Video Games Debate. Eur J Soc Psychol. 2014;(3):104–16.

[15] Nauroth P, Gollwitzer M, Bender J, Rothmund T. Social identity threat motivates science-discrediting online comments. PLoS One [Internet]. 2015;10(2). Available from: http://dx.plos.org/10.1371/journal.pone.0117476 10.1371/journal.pone.0117476 PMC4315604 25646725

[16] Honneth A. The struggle for recognition: The moral grammar of social conflicts. Cambridge, UK: Polity Press; 1995.

[17] Simon B, Grabow H. To be respected and to respect: the challenge of mutual respect in intergroup relations. Br J Soc Psychol [Internet]. 2014;53:39–53. Available from: http://www.ncbi.nlm.nih.gov/pubmed/24627991 10.1111/bjso.12019 24627991

[18] Simon B, Grabow H, Böhme N. On the meaning of respect for sexual minorities: the case of gays and lesbians. Psychol Sex [Internet]. Routledge; 2015;6(4):297–310. Available from: http://www.tandfonline.com/doi/full/10.1080/19419899.2014.987683

[19] Huo YJ, Molina LE. Is Pluralism a Viable Model of Diversity? The Benefits and Limits of Subgroup Respect. Gr Process Intergr Relations. 2006;9(3):359–76.

[20] Smith HJ, Tyler TR. Choosing the right pond: The impact of group membership on self-esteem and group-oriented behavior. J Exp Soc Psychol. 1997;33(2):146–70.

[21] Simon B, Schaefer CD. Tolerance as a function of disapproval and respect: The case of Muslims. Br J Soc Psychol. 2016;55(2):375–83. 10.1111/bjso.12137 26670416

[22] Simon B, Schaefer CD. Muslims’ tolerance towards outgroups: Longitudinal evidence for the role of respect. Br J Soc Psychol. 2018;57(1):240–9. 10.1111/bjso.12213 28815636

[23] Bendersky C. Resolving ideological conflicts by affirming opponents’ status: The Tea Party, Obamacare and the 2013 government shutdown. J Exp Soc Psychol. 2014;53:163–168.

[24] Leach CW, Zomeren M, Zebel S, Vliek ML, Pennekamp SF, Doosje B, et al Group-level self-definition and self-investment: a hierarchical (multicomponent) model of in-group identification. J Pers Soc Psychol [Internet]. 2008;95(1):144–65. Available from: 10.1037/0022-3514.95.1.144 18605857

[25] Simon B, Mommert A, Renger D. Reaching across group boundaries: Respect from outgroup members facilitates recategorization as a common group. Br J Soc Psychol. 2015;54. 10.1111/bjso.12112 25879772

[26] Aiken LS, West SG. Multiple regression: Testing and interpreting interactions. Newbury Park, CA: Sage Publications; 1991.

[27] Carpini MXD, Cook FL, Jacobs LR. Public deliberation, discursive participation, and citizen engagement: A review of the empirical literature. Annu Rev Polit Sci [Internet]. 2004;7(1):315–44. Available from: http://www.annualreviews.org/doi/10.1146/annurev.polisci.7.121003.091630

[28] Postmes T. Psychology: Climate change and group dynamics. Nat Clim Chang [Internet]. 2015;5(3):195–6. Available from: http://www.nature.com.ezproxy.canterbury.ac.nz/nclimate/journal/vaop/ncurrent/full/nclimate2537.html%5Cnhttp://www.nature.com/doifinder/10.1038/nclimate2537

[29] Skitka LJ, Houston DA. When Due Process Is of No Consequence: Moral Mandates and Presumed Defendant Guilt or Innocence. Soc Justice Res. 2001;14(3):305–26.

[30] Skitka LJ, Morgan GS. The social and political implications of moral conviction. Polit Psychol. 2014;35(SUPPL.1):95–110.

[31] Branscombe NR, Wann DL. Collective self-esteem consequences of outgroup derogation when a valued social identity is on trial. Eur J Soc Psychol. 1994;24(6):641–57.

[32] Rothgerber H. External intergroup threat as an antecedent to perceptions in in-group and out-group homogeneity. J Pers Soc Psychol. 1997;73(6):1206–12. 9418276 10.1037//0022-3514.73.6.1206

[33] Tajfel H, Turner JC. The social identity theory of intergroup behavior In: Worchel S, Austin WG, editors. Psychology of intergroup relations. 2nd ed Chicago, IL: Nelson- Hall; 1986 p. 7–24.

[34] Riek BM, Mania EW, Gaertner SL. Intergroup threat and outgroup attitudes: A meta-analytic review. Personal Soc Psychol Rev [Internet]. 2006;10(4):336–53. Available from: http://journals.sagepub.com/doi/10.1207/s15327957pspr1004_4 10.1207/s15327957pspr1004_4 17201592

[35] Iyengar S, Sood G, Lelkes Y. Affect, not ideology: A social identity perspective on polarization. Public Opin Q. 2012;76(3):405–31.

[36] Mason L. “I disrespectfully agree”: The differential effects of partisan sorting on social and issue polarization. Am J Pol Sci. 2015;59(1):128–45.

[37] Simon B. A realistic view on disagreement: Roots, resolutions, and the trauma of the scientist. Theory Psychol. Forthcoming.

