# Supplementary material for: Respect and political disagreement: Can intergroup respect reduce the biased evaluation of outgroup arguments?
Source: PLoS One. 2019 Mar 26;14(3):e0211556. doi: 10.1371/journal.pone.0211556 (PMC6435108; doi:10.1371/journal.pone.0211556)
Supplement: S1 Appendix — In this pdf document results for Study 1 are presented in tables. (PDF) [file pone.0211556.s001.pdf]

Table A. Study 1: Descriptive Statistics.

Variable		M	SD	1)	2)	3)	4)	5)	6)	7)
1)	Sex									
2)	Argument position									
3)	Ingroup identification (proponents)	3.85	1.06	.03	--	.70				
4)	Ingroup identification (opponents)	3.95	1.58	.08	--	--	.80			
5)	Pro arguments	4.53	1.12	.28**	.59**	.39**	-.39*	.90		
6)	Con arguments	4.23	1.02	.02	-.42**	-.33**	.33*	-.43**	.88	
7)	Arguments total	4.38	0.57	.29**	.21**	.01	-.09	--	--	.75

Note. Correlations significant at ***p* < .01, **p* < .05; Cronbach's alpha are in diagonal cells.

Table B. Study 2: Linear Mixed Models with Argument Position, Participant Position and Ingroup Identification.

Variable	*df*	*F*	*p*	*ηp²*
Argument position	1, 99	0.08	.784	< .001
Participant position	1, 97.03	2.45	.121	.02
Ingroup Identification	1, 97	0.35	.852	.003
sex	1, 97	6.49	.002	.06
Participant position x Argument position	1, 99	68.33	< .001	.41
Argument position x ingroup Identification	1, 99	0.53	.467	.005
Participant position x ingroup Identification	1, 97	0.05	.824	< .001
Participant position x argument position x ingroup Identification	1, 99	25.58	< .001	.21

Table C. Study 2: Linear Mixed Models with Argument Position, Participant Position and Ingroup Identification (at 1 SD above the mean).

Variable	*df*	*F*	*p*	*ηp²*
Argument position	1, 99	0.50	.480	.005
Participant position	1, 97,07	1.61	.208	.02
Ingroup Identification	1, 97	0.04	.852	< .001
sex	1, 97	6.49	.002	.06
Participant position x Argument position	1, 99	89.76	< .001	.47
Argument position x ingroup Identification	1, 99	0.53	.467	.005
Participant position x ingroup Identification	1, 97	0.05	.824	< .001
Participant position x argument position x ingroup Identification	1, 99	25.58	< .001	.21

Table D. Study 2: Linear Mixed Models with Argument Position, Participant Position and Ingroup Identification (at 1 SD below the mean).

**Variable**	***df***	***F***	***p***	***ηp²***
Argument position	1, 99	0.10	.756	< .001
Participant position	1, 96,97	0.95	.331	.009
Ingroup Identification	1, 97	0.04	.852	< .001
sex	1, 97	6.49	.002	.06
Participant position x Argument position	1, 99	5.67	.019	.05
Argument position x ingroup Identification	1, 99	0.53	.467	.005
Participant position x ingroup Identification	1, 97	0.05	.824	< .001
Participant position x argument position x ingroup Identification	1, 99	25.58	< .001	.20
