# Supplementary material for: Respect and political disagreement: Can intergroup respect reduce the biased evaluation of outgroup arguments?
Source: PLoS One. 2019 Mar 26;14(3):e0211556. doi: 10.1371/journal.pone.0211556 (PMC6435108; doi:10.1371/journal.pone.0211556)
Supplement: S2 Appendix — In this pdf document results for Study 2 are presented in tables. (PDF) [file pone.0211556.s002.pdf]

Table E. Study 2: Descriptive Statistics.

Variable		M	SD	1)	2)	3)	4)
1)	Sex						
2)	Ingroup identification (proponents)	3.81	1.08	.02	.66		
3)	Ingroup identification (opponents)	4.28	1.14	-.21	--	.74	
4)	Outgroup arguments	3.90	0.94	.34**	.03	-.18	.73 / .82

Note. Correlations significant at ***p* < .01; Cronbach's alpha are in diagonal cells.

Table F. Study 2: Manipulation Checks.

Variable		*df*	*F*	*p*	*ηp²*
Participant position	individual equality recognition	1,133	1.90	.171	.01
	group equality recognition	1,133	3.42	.067	.03
Respect	individual equality recognition	1,133	235.25	< .001	.64
	group equality recognition	1,133	280.90	< .001	.68
Participant position x respect	individual equality recognition	1,133	.003	.955	< .001
	group equality recognition	1,133	< .001	.988	< .001

Table G. Study 2: Multiple Linear Regression Model with Participant Position, Induced Respect and Ingroup Identification.

Variable	*B*	*SE*	*p*
Participant position	.08	.08	.289
Respect	.03	.08	.707
Ingroup identification	-.02	.07	.832
Sex	.64	.15	< .001
Participant position x respect	-.02	.08	.802
Participant position x ingroup Identification	.09	.07	.218
Respect x ingroup Identification	.18	.07	.016
Participant position x respect x ingroup Identification	-.02	.07	.763
